# Phosphatidylcholine-specific phospholipase C inhibition down- regulates CXCR4 expression and interferes with proliferation, invasion and glycolysis in glioma cells

**DOI:** 10.1371/journal.pone.0176108

**Published:** 2017-04-19

**Authors:** Laura Mercurio, Serena Cecchetti, Alessandro Ricci, Aurora Pacella, Giovanni Cigliana, Giuseppina Bozzuto, Franca Podo, Egidio Iorio, Giulia Carpinelli

**Affiliations:** 1 Department of Cell Biology and Neurosciences, Istituto Superiore di Sanità, Rome, Italy; 2 Clinical Pathology Laboratories, Department of Research, Advanced Diagnostics, and Technological Innovation, Translational Research Area, Regina Elena National Cancer Institute, Rome, Italy; 3 Department of Technology and Health, Istituto Superiore di Sanità, Rome, Italy; Norges teknisk-naturvitenskapelige universitet, NORWAY

## Abstract

**Background:**

The chemokine receptor CXCR4 plays a crucial role in tumors, including glioblastoma multiforme (GBM), the most aggressive glioma.

Phosphatidylcholine-specific phospholipase C (PC-PLC), a catabolic enzyme of PC metabolism, is involved in several aspects of cancer biology and its inhibition down-modulates the expression of growth factor membrane receptors interfering with their signaling pathways.

In the present work we investigated the possible interplay between CXCR4 and PC-PLC in GBM cells.

**Methods:**

Confocal microscopy, immunoprecipitation, western blot analyses, and the evaluation of migration and invasion potential were performed on U87MG cells after PC-PLC inhibition with the xanthate D609. The intracellular metabolome was investigated by magnetic resonance spectroscopy; lactate levels and lactate dehydrogenase (LDH) activity were analyzed by colorimetric assay.

**Results:**

Our studies demonstrated that CXCR4 and PC-PLC co-localize and are associated on U87MG cell membrane. D609 reduced CXCR4 expression, cell proliferation and invasion, interfering with AKT and EGFR activation and expression. Metabolic analyses showed a decrease in intracellular lactate concentration together with a decrement in LDH activity.

**Conclusions:**

Our data suggest that inhibition of PC-PLC could represent a new molecular approach in glioma biology not only for its ability in modulating cell metabolism, glioma growth and motility, but also for its inhibitory effect on crucial molecules involved in cancer progression.

## Introduction

Glioblastoma multiforme (GBM), the most aggressive and frequent glioma which represents about 50% of all brain tumors, is characterized by an aberrant network of molecular signaling pathways that drive uncontrolled cell proliferation, high invasivity, aberrant angiogenesis and high cellular heterogeneity [1].

Among the factors recently described to be implicated in different biological features of gliomas, an increasing attention has been focused on some chemokine/chemokine receptor axes. Among these, the system formed by the chemokine receptor CXCR4 and its cognate ligand, the chemokine SDF-1α/CXCL12, has been highlighted to play a crucial role in multiple mechanisms sustaining tumor progression [2–4].

CXCR4 is a transmembrane G-protein-coupled receptor, widely expressed in several tumor types, whose binding with the chemokine CXCL12 results in the activation of down-stream signal transduction pathways such as the phosphorylation of mitogen activated protein (MAP) kinases and AKT-mediated signaling, responsible for multiple features of malignancy including chemotaxis, cell survival, cell proliferation, increased intracellular calcium and transcription of genes involved in angiogenesis, inflammation and metastasis [5, 6].

Several reports demonstrated a correlation between high levels of CXCL12 and CXCR4 and tumor malignancy in different tumor types, including gliomas [7]. Numerous studies on preclinical glioma models have shown that the blockade of the CXCL12/CXCR4 axis by specific antagonists affects tumor growth, vasculogenesis and post-radiation recurrence [8–10]. Furthermore, using an *in vitro* and *in vivo* U87MG glioma model, we recently showed that CXCR4 inhibition with a novel peptide antagonist not only induces alterations in molecular responses strictly related to the tumor cells but also modulates the reactivity of glioma-associated microglia/macrophages (GAMs), with a polarization into a proinflammatory GAMs phenotype that could be correlated with a potential anti-tumor activity [11].

Despite numerous encouraging results obtained using CXCR4-targeting treatments in preclinical models, to date this approach has not shown a strong efficacy against GBM growth and progression. The complex biology of GBM and the existence of redundancy and co-activation of multiple molecular pathways require the use of novel strategies for treatment of GBM patients.

Previous studies by our group showed that a catabolic enzyme of the phosphatidylcholine (PC) cycle, the PC-specific phospholipase C (PC-PLC), is crucially involved in multiple aspects of cancer biology such as cell metabolism, proliferation, survival and differentiation [12–20]. PC-PLC catalyses the hydrolysis of PC by producing phosphocholine (PCho) and 1,2-diacylglycerol (DAG), a second messenger that induces the activation of protein kinase C (PKC) which in turn phosphorylates several proteins involved in multiple transduction cascades.

Interestingly, the inhibition of PC-PLC by a competitive inhibitor (D609) down-modulates the expression of some growth factor membrane receptors in breast cancer cells [16] thus interfering with cell receptor-activated signal transduction pathways involved in tumor progression [20].

On the basis of these previous data, pointing to links between PC-PLC enzyme activation and growth factor receptors’ status and in light of our previous study on CXCR4 in U87MG glioma cells [11], in the present work we investigated the possible cross-talk between CXCR4 and PC-PLC in U87MG cells using the competitive PC-PLC inhibitor D609 [21] and compared the effects of this agent with those exerted by a conventional CXCR4 antagonist (Plerixafor).

## Materials and methods

### Chemicals

All chemicals were purchased from Life Technologies (Carlsbad, VA, USA) unless otherwise specified. D609 (tricyclodecan-9-yl-xanthogenate) and Plerixafor were supplied by Sigma Aldrich (St Louis, MO, USA).

### Antibodies and reagents

Rabbit polyclonal antibody raised against bacterial (*Bacillus cereus*) PC-PLC and selectively cross-reacting with a mammalian PC-PLC isoform (66 kDa) was obtained in our laboratory according to Clark *et al* [22] and characterized as previously described [23, 24]. The following antibodies were used: mouse anti-human CXCR4 monoclonal antibody (R&D Systems, Minneapolis, MN, USA, cat. n° MAB172, 1:50), rabbit polyclonal anti-CXCR4 (Sigma-Aldrich, cat. n°C8227, 1:1000), mouse monoclonal anti-β-actin (Sigma-Aldrich, cat. n°A5441, 1:2000), rabbit polyclonal anti-phosphorylated AKT (Ser473, Cell Signaling, cat. n°9271, 1:1000), rabbit polyclonal anti-AKT (Cell Signaling, Danvers, MA, USA, cat. n°9272, 1:1000), mouse monoclonal anti-phospho-MAPK (ERK1/2, Thr202/Tyr204, Cell Signaling, cat. n°9106, 1:1000), rabbit polyclonal anti-MAPK (ERK1/2, Sigma-Aldrich, cat n°M5670, 1:10000), mouse monoclonal anti-phospho-EGFR (Tyr1068, Cell Signaling, cat. n°2236, 1:500), rabbit monoclonal anti-EGFR (Cell Signaling, cat. n°4267, 1:1000).

The secondary antibodies Alexa Fluor-594 or -488- F(ab’)_2_ fragments of goat anti-rabbit (cat. n° A-11072, 1:200) and goat anti-mouse (cat. n° A-11017, 1:200) were purchased from Molecular Probes (Life Technologies); horseradish peroxidase-conjugated goat anti-mouse IgG (cat. n° 170–6516, 1:3000) and goat anti-rabbit IgG (cat. n° 170–6515, 1:3000) were from BioRad Laboratories Inc.

### Cells

The human glioblastoma multiforme (GBM) cell line U87MG, purchased from American Type Culture and Collection (ATCC, Manassas, VA, USA) was cultured in MEM at 5% CO2 and 95% humidified atmosphere air at 37°C. The medium was supplemented with 10% fetal bovine serum (Sigma-Aldrich), 2mM glutamine, 100 μg/ml streptomycin, 100 U/ml penicillin and 1mM sodium pyruvate.

### Cell treatments

U87MG cells were seeded at a density of 4.0 x 10^4^ cells per cm^2^, cultured in complete medium at 37°C for 24h and then incubated in absence (CTRL) or presence of either 10 μM Plerixafor, a CXCR4 antagonist [11], or with the competitive PC-PLC inhibitor, tricyclodecan-9-yl-potassium xanthate (D609). Both inhibitors were suspended in PBS and were added to cells with an equal volume of fresh medium as for control condition. The sensitivity of U87MG cells to D609 was evaluated by 3-(4,5-dimethylthiazol-2-yl)-2,5-diphenyl tetrazolium bromide (MTT) assay, as previously described [16, 25] on cells exposed to increasing concentrations of D609, from 20 μM to 300 μM for 48h (S1 Fig). For the subsequent experiments, we utilized 100 μM concentration of D609, which inhibited U87MG cell proliferation by 50.0 ± 0.3% at 48h of cell exposure (P (treated versus untreated) ≤ 0.001) without inducing any significant alteration in cell viability (≥ 90% at any tested time of exposure to the drug).

### Cell proliferation of treated cells

The effects exerted by the PC-PLC inhibitor and the CXCR4 antagonist on U87MG cell proliferation were investigated using Trypan blue exclusion tests at different time points (24h, 48h and 72h).

### Confocal laser scanning microscopy (CLSM)

CLSM experiments were conducted on non-permeabilized U87MG cells in order to investigate the localization and distribution of CXCR4 and PC-PLC on plasma membrane. GBM cells were plated in 24-well plates onto 12-mm cover glasses and treated for 24, 48, 72h as described in the “Cell treatments” section.

Prior to fixation cells were incubated with either anti-PC-PLC (1:50) or anti-CXCR4 antibodies (1:50). Alexa Fluor^®^ 488-F(ab’)_2_ fragments of goat anti-mouse, and -594 F(ab’)_2_ fragments of goat anti-rabbit, were used as secondary Abs. After extensive washing in PBS and fixation with methanol, the coverslips were mounted with Vectashield® antifade mounting medium containing DAPI (Vector Labs, Burlingame, CA, USA).

The examinations were performed on a Leica TCS SP2 AOBS apparatus (Leica, Wetzlar, Germany) as previously reported [11].

### Transwell chamber migration and invasion assays

The effects of D609 on migration and invasive potential of U87MG cells were analyzed by a transwell chamber [26] assay by using inserts with 8.0-μm pore (BD Biosciences, Sparks, MA, USA) which stood in six-well plates. The procedure for carrying out invasion and migration assays was the same described previously [18]. U87MG cells were plated and treated as previously described and after 24h of treatment cells were harvested and seeded in the inner side of the transwell chamber in serum-free medium in the absence of the inhibitors. U87MG cells were allowed to migrate for 20 h at 37°C. The detection and analysis of cells that migrated and invaded through the membrane was performed as described previously [18].

### Western blotting (WB)

U87MG cells were seeded and treated with D609 or Plerixafor as described above. Protein expression was evaluated in total cell lysates (30 μg of proteins) processed as previously described [11] and blotted with the antibodies (Abs) described in “Antibodies and reagents” section. Horseradish peroxidase (HRP)-conjugated antibodies, goat anti-mouse and goat anti-rabbit were used as secondary antibodies. Immobilion^TM^ Western reagents (Merck Millipore, Billerica, MA, USA) were used as chemoluminescent substrates. Images were acquired and elaborated by a Multispectral Imaging system UVP (Biospectrum, Upland, CA, USA). Densitometric analyses were performed using Image J software (imagej.nih.gov/ij/).

### Immunoprecipitation

U87MG cells were prepared for immunoprecipitation tests as previously described [27]. In brief, total cell lysates (1 mg/ml) were incubated with 10% protein G Sepharose (Amersham Biosciences, Uppsala, Sweden) in the presence of anti-CXCR4 Ab overnight at 4°C. After extensive washing, beads were removed by centrifugation at 14,000 rpm and then SDS sample buffer 4×, containing 2-mercaptoethanol (ICN Biomedicals Inc., Irvine, CA, USA) was added to elute proteins by heating the sample at 100°C for 5 minutes. Immunoprecipitates were resolved by 7% SDS-PAGE under reducing conditions and blotted with anti-CXCR4 and/or anti-PC-PLC Abs.

### Proton magnetic resonance spectroscopy

High-resolution proton magnetic resonance spectroscopy (^1^H MRS) analyses were performed at 25°C using a 400 MHz Bruker Avance spectrometer (9.4 Tesla) (Bruker, Karlsruhe, Germany) on U87MG cells treated for 24h, 48h and 72h as described in the “Cell treatments” section.

At each time point of treatment, GBM cells were harvested, washed with cold PBS and processed for MRS experiments as previously described [13]. U87MG ethanolic cell extracts were prepared by adding 70% of ethanolic solution (EtOH:H2O, 70:30 v/v) to cells. Samples were sonicated at 20 kHz by a MSE ultrasonic disintegrator Mk2 (exponential probe, 8 μm peak to peak) and centrifuged at 3,000 rpm for 30 minutes. The supernatants were lyophilised in a RVT 4104 Savant lyophiliser, and the residue resuspended in D_2_O (Sigma-Aldrich) containing 0.1 mM trimethylsilyl-propionic-2,2,3,3-d4 acid sodium salt (TSP) using trimethyl-silyl-propionic-2,2,3,3-d4 acid (TSP) sodium salt as chemical shift and peak area reference (Merck & Co,Montreal, Canada).

^1^H MR spectra of cell extracts were obtained using acquisition pulses, water presaturation, data processing, and data analysis according to an established protocol developed in our laboratory [28, 29] using a 1mm TXI Microprobe. Spectra of extracts were zero-filled by doubling the number of data points (to 32 K) and line-broadened by 0.5 Hz prior to Fourier transformation and subsequently quantitated by integration using WINNMR Bruker software and/or Topspin 3.0. The latter allowed baseline correction by application of a cubic splines function through appropriate points. All spectra were repeated on standard compounds for verification of signal assignments, which were further confirmed by literature reports.

Relative metabolite contents (C(m)) were determined by measuring the area (integral) of the signals of the corresponding metabolite chemical groups (I(mcg)) referring to the integral of the reference standard (I(r)). Furthermore, we referred each integral to the number of equivalent protons in the metabolite chemical group (N(mcg)) and in the reference standard molecule (N(r)), applying the equation:

C(m) = [[I(mcg)] /[I(r)] ×[N(r)] /[N(mcg)]].

Metabolite quantification was expressed as metabolite percentage versus the total metabolites.

For peak assignment of “total choline” (tCho) resonance band samples were analyzed using a 700 MHz Bruker Avance spectrometer (16.4 Tesla) (Bruker, Karlsruhe, Germany). Choline containing metabolites have been identified by an increase of proton ^1^HMRS resonance band at 3.2 ppm, due to the trimethylammonium (─N(CH)3)3 headgroups of phosphocholine (PCho), glycerophosphocholine (GPC) and free choline (Cho).

### Determination of lactate content and lactate dehydrogenase activity

Cells were seeded and treated as described in the previous sections. After 24h, 48h and 72h of treatment U87MG-conditioned medium was collected and cells were harvested and lysed on ice for 30 minutes with a lysis buffer [0.2M Tris HCl pH 8; 0.1% Triton X-100; 0.09% NaCl] then centrifuged at 13,000 rpm at 4°C for 10 minutes. The extracellular lactate concentration was measured in supernatants, while intracellular lactate and lactate dehydrogenase (LDH) activity were measured in cell lysates using diagnostics assay Kit CE-IVD approved on instrumentation Roche/Hitachi Cobas C System: LACT colorimetric assay (Roche cat. n°03183700–190) and LDH enzymatic assay (Roche cat. n° 20767123–322).

### Statistical analysis

Statistical analyses were performed using GraphPad Prism 4 (GraphPad Software Inc.). Statistical significance of differences was determined by two-tailed unpaired Student t-Test or one-way ANOVA as specified. Differences were considered significant when P < 0.05.

## Results

### Co-localization and association of PC-PLC with CXCR4 in U87MG cells

**Fig 1:**
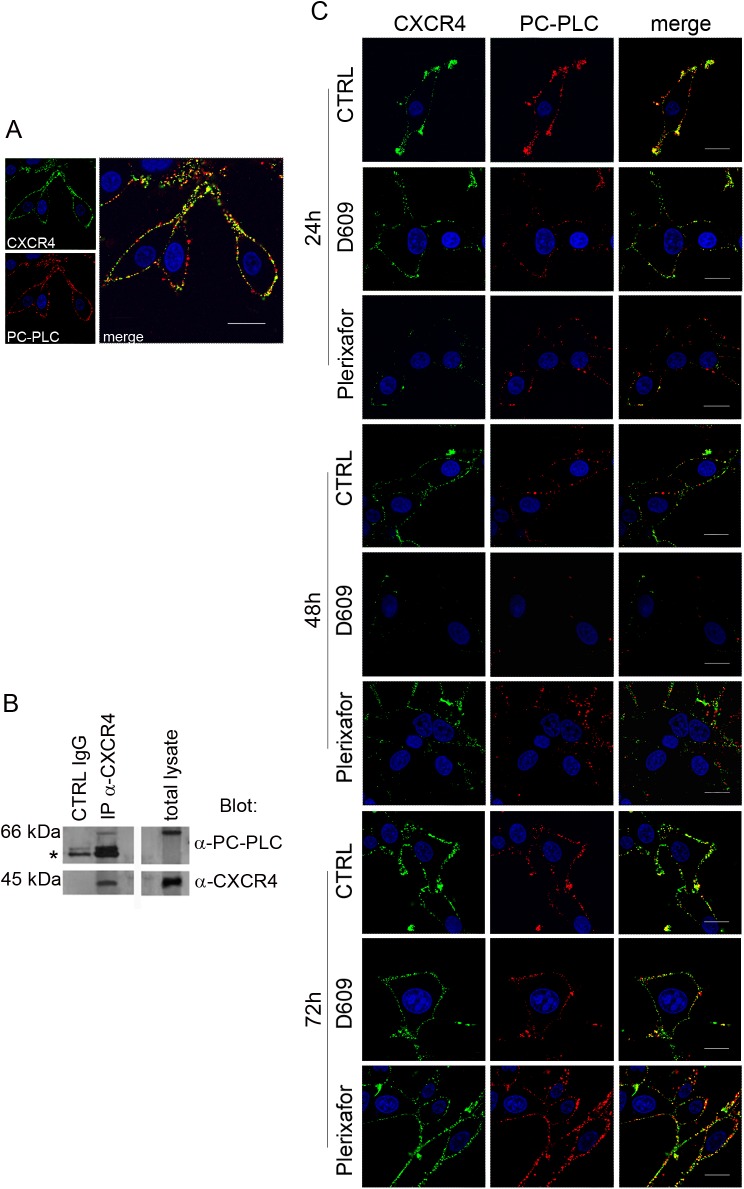
CXCR4 and PC-PLC expression on U87MG cell. A) Confocal laser scanning microscopy (CLSM) analyses of CXCR4 (green fluorescence) and PC-PLC (red fluorescence) localization on plasma membrane of unfixed cells. Scale bars, 18μm. B) Western blot (WB) of proteins isolated from U87MG cells by immunoprecipitation with anti-CXCR4 Ab (IP-α-CXCR4). Top panels show IP-α-CXCR4 preparations blotted with anti-PC-PLC Ab compared to control (CTRL IgG) (left) and total cell lysate (right). *IgG heavy chains. The bottom panels represent an α-CXCR4-IP preparation blotted with anti-CXCR4 Ab compared with CTRL IgG (left) and total lysate (right). C) CLSM analyses of CXCR4 (green fluorescence) and PC-PLC localization (red fluorescence) on plasma membrane of unfixed U87MG cells following the respective treatments (indicated on the left of the panel rows). Images represent untreated cells (CTRL) and cells exposed to either D609 or Plerixafor at different time-points (24h, 48h, 72h). Scale bars, 16 μm.

CLSM analyses highlighted for the first time an extensive co-localization of PC-PLC and CXCR4 on the plasma membrane of unfixed cells, as reported in Fig 1A. Moreover, WB of proteins immunoprecipitated with anti-CXCR4 Abs showed the presence of a distinct protein band due to PC-PLC (66 kDa), confirming the existence of an association between PC-PLC and CXCR4 in U87MG cells (Fig 1B).

### Effects of PC-PLC inhibition on CXCR4 expression

The localization of PC-PLC and CXCR4 on plasma membrane was evaluated by CLSM analyses of non-permeabilized U87MG cells exposed for different time intervals (24h, 48h, 72h) either to the PC-PLC inhibitor D609 (100 μM) or to the CXCR4 antagonist Plerixafor (10 μM). Plerixafor strongly decreased the CXCR4 expression on cell membrane of U87MG cells treated for 24h compared to untreated (CTRL) cells (Fig 1C, green fluorescence) as already reported [11]. However, at 48h of treatment with this antagonist the CXCR4 receptor was already re-expressed on cell membrane and its level was further increased at 72h (Fig 1C). The PC-PLC inhibitor also induced a substantial decrease in the CXCR4 membrane expression at 24h, an effect which persisted at 48h, while at 72h the receptor was re-expressed on membrane to a level similar to that of untreated cells.

**Fig 2:**
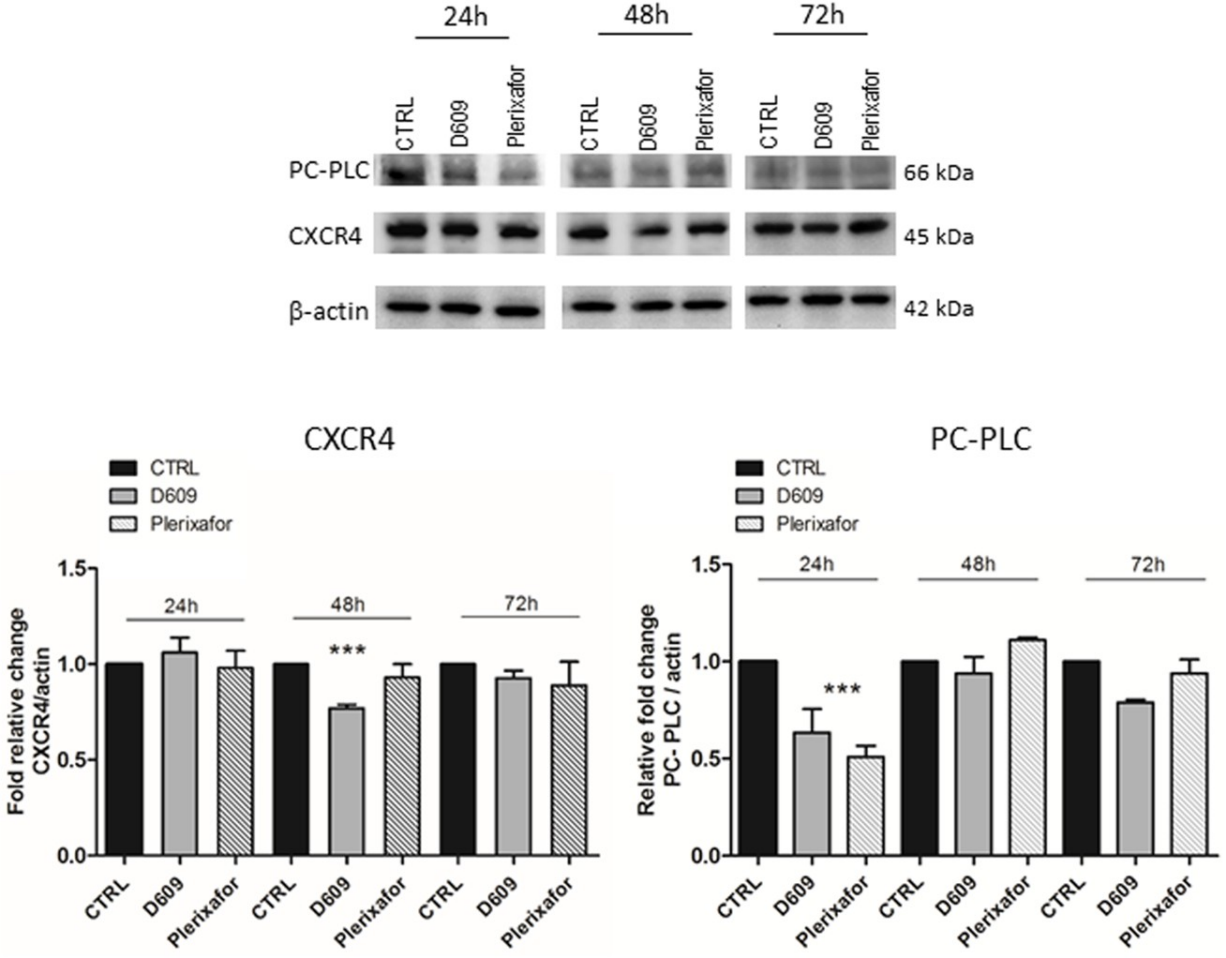
Analysis of total CXCR4 and PC-PLC protein contents in U87MG cell lysates. Representative WB of CXCR4 and PC-PLC detection in U87MG cells processed after 24h, 48h and 72h of treatment with either D609 or Plerixafor. β-actin was used as loading control. The histograms represent the mean value ± SD of relative fold change of CXCR4 (on the left) and PC-PLC (on the right) optical density (OD) normalized to β-actin, obtained by densitometric analyses of WB bands (Image J software). CTRL values normalized to 1 at each time point; n = 3 independent experiments. Statistical analyses were performed with one-way ANOVA *** P = 0.0008 (CXCR4 expression), ***P = 0.0008 (PC-PLC expression).

WB experiments on total cell lysates and the relative densitometric analyses showed that D609 (but not Plerixafor) induced a significant decrease in the total cellular CXCR4 protein content with a reduction of about 24% at 48h of treatment, compared to CTRL and Plerixafor-treated U87MG cells (Fig 2).

We also observed a strong decrease of the PC-PLC localized on plasma membrane of cells exposed to D609 (Fig 1C, red fluorescence, D609), as already reported for other cancer cell models [15]. This effect, already evident after 24h of treatment, further increased at 48h, where the enzyme was almost undetectable on cell membrane. At 72h of D609-treatment PC-PLC was re-expressed on membrane, but to a lower intensity compared to both CTRL and Plerixafor treated cells. Interestingly, the CXCR4 antagonist was also able to down-modulate the PC-PLC expression on U87MG cell membrane, the maximum effect being detected at 24h of treatment (Fig 1C), thus supporting the hypothesis of a cross-talk between this phospholipase and the receptor on the plasma membrane of these cells. Although PC-PLC expression significantly decreased over time during cell growth in untreated U87MG cells (up to 50% ± 0.05 at 72h), quantitative analyses of WB experiments showed that both agents were further able to significantly reduce the PC-PLC content by 40% in D609- and 50% in Plerixafor-treated cells at 24h as compared to the corresponding untreated controls (Fig 2).

### Effects of D609 on cell growth, migration and invasion

**Fig 3:**
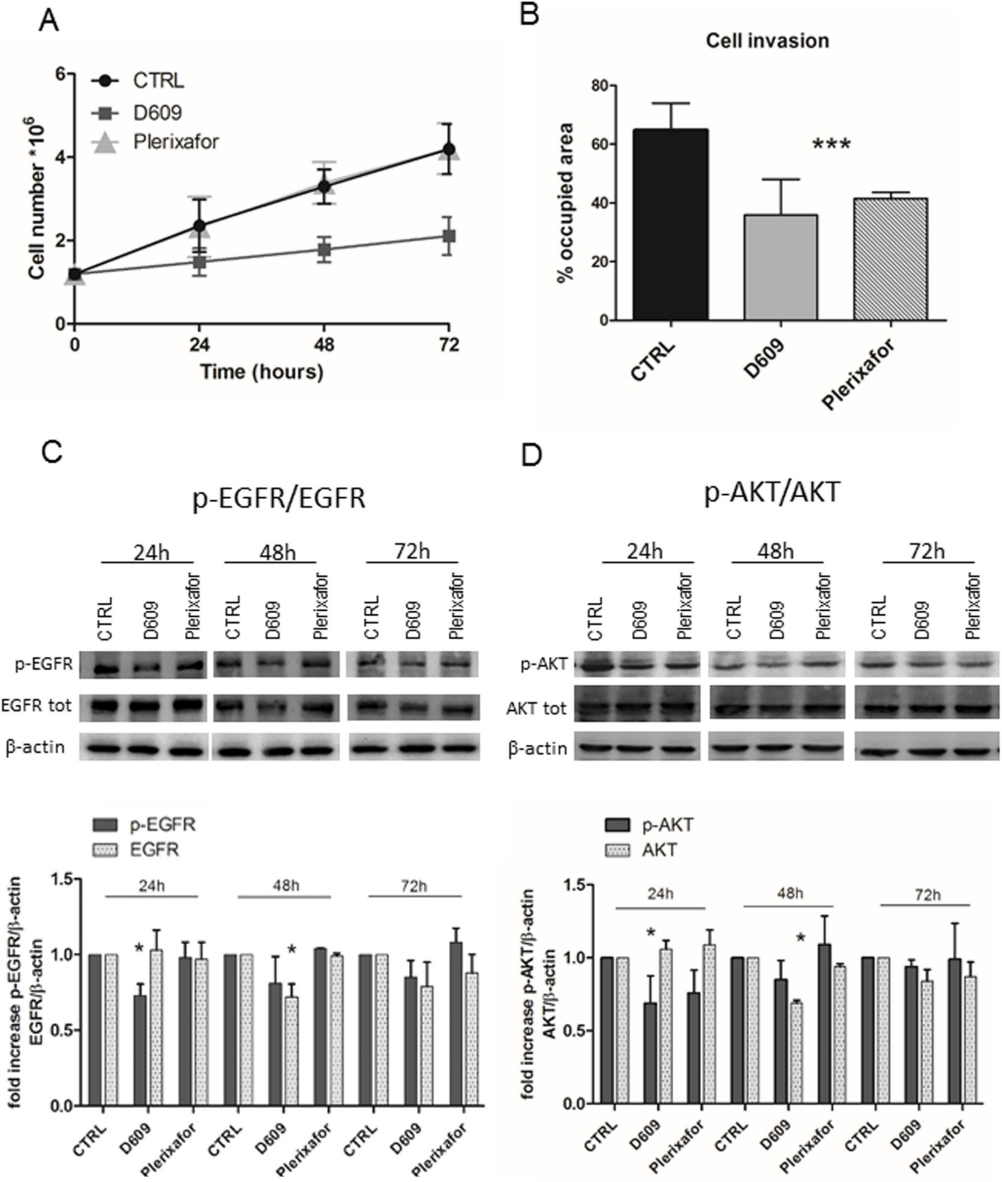
Effects of D609 and Plerixafor on cell proliferation, invasion and cell signaling. A) U87MG cell proliferation after 24h, 48h, 72h of treatments with D609 or Plerixafor evaluated by cell counting using the Trypan blue excluding assay. Curves represent mean values ± SD of U87MG cell counts at different time-points (n = eight independent experiments). ● CTRL; ■ (dark grey square) D6099; and ▲ (light grey triangle) Plerixafor. Statistical analyses were performed with two-tailed unpaired t Student’s test. CTRL versus D609: 24h * P = 0.05; 48h *** P = 0.0001; 72h ** P = 0.0005. B) U87MG cell invasion evaluated by transwell chamber assay using Matrigel, in response to D609 or Plerixafor 24h treatment. The histograms represent the percentage of the area occupied by U87MG cells. CTRL values = 100%. Mean values ± SD (n = 6). Statistical analyses were performed with one-way ANOVA. ***P = 0.0001. C) Representative WB of p-EGFR and total EGFR expression in U87MG cells either untreated (CTRL), or treated with D609 or with Plerixafor for 24h, 48h and 72h. β-actin was used as loading control. The histograms represent the mean values of relative fold changes of p-EGFR and EGFR optical density normalized to β-actin, obtained with densitometric analyses of the WB protein bands (Image J software). CTRL values = 1. Means ± SD of n = 3 independent experiments. Statistical analyses were performed with one-way ANOVA * P = 0.0333 (p-EGFR); * P = 0.0141 (EGFR). D) Representative WB of p-AKT and AKT expression in U87MG control cells (CTRL) and in cells treated with either D609 or Plerixafor for 24h, 48h and 72h. β-actin was used as loading control. Histogram represents the mean of relative fold change of p-AKT and AKT optical density normalized to β-actin, obtained with densitometric analyses of WB protein bands (Image J software). CTRL values = 1. Means ± SD of n = 3 independent experiments. Statistical analyses were performed with one-way ANOVA * P = 0.0230 (p-AKT); * P = 0.0165 (AKT).

We investigated the changes in cell growth and cell motility in response to either D609 or Plerixafor treatment. U87MG cell proliferation was monitored in cells after 24h, 48h and 72h of treatment using Trypan blue exclusion tests. These analyses reported a significant inhibitory effect on proliferation after cell exposure to D609 (with average decreases in cell count of 37% at 24h; 46% at 48h; and 40% at 72h) compared to either untreated (CTRL) or Plerixafor–treated cells (Fig 3A). The most significant anti-proliferative effect was observed at 48h of D609 treatment. No significant cell death, detected by Trypan blue exclusion assay, was induced by either agent at any time. Cell invasion analyses showed that D609 was able to induce a stronger inhibition of U87MG cell invasion (46%) compared with that induced by the conventional CXCR4 antagonist (38%) (Fig 3B), which proved to inhibit glioma cell motility [8,30]. Neither D609 nor Plerixafor induced significant effects on U87MG cell migration (data not shown).

### PC-PLC inhibition reduces the p-EGFR/EGFR and p-AKT/AKT expression in U87MG cells

To further investigate the effects of D609 and Plerixafor on U87MG cells we performed WB analyses to evaluate the expression of p-EGFR and total EGFR after 24h, 48h and 72h of treatment with these agents. The experiments showed that D609, but not Plerixafor, induced a significant decrease (by 27%) in the level of EGFR phosphorylation in cells treated for 24h compared with the untreated (CTRL) cells (Fig 3C). Interestingly, D609 also induced a reduction (by 28%) in the total EGFR expression level after 48h of treatment compared to CTRL and Plerixafor-treated cells (Fig 3C). At 72h D609-treated cells had a tendency to maintain lower levels of total EGFR in comparison with Plerixafor-treated and untreated cells. Furthermore, WB analysis of anti-CXCR4 immunoprecipitates from total cell lysates, blotted with anti-EGFR Ab highlighted an association between the two receptors in U87MG cells (S2 Fig).

In order to further investigate how treatments with either D609 or Plerixafor could interfere with the CXCR4 and PC-PLC-mediated signal transduction pathways, we evaluated the expression of two key kinases such as AKT and ERK1/2, at different times of treatment. WB analyses showed that both D609 and Plerixafor reduced the activation of AKT at 24h, although a more pronounced effect (about 30%) was induced by the former agent. Moreover, a reduction of about 30% was also detected in the total content of AKT in cells exposed for 48h to D609 compared with CTRL or Plerixafor-treated cells (Fig 3D) suggesting a possible effect of the PC-PLC inhibitor on protein synthesis. At 72h no significant changes in the expression of p-AKT and total AKT protein expression levels could be detected.

No changes were induced on the p-ERK or total ERK protein expression levels in cells treated with either D609 or Plerixafor at any time point (S3 Fig).

### Changes in cell metabolism of U87MG glioma cells exposed to D609

It is worth noting that PC-PLC inhibition by D609 induces alterations in PC metabolism in a variety of cancer cells [14]. In the present work we performed ^1^H MRS analyses on ethanolic extracts of U87MG cells in order to evaluate the effects of treatment on cell metabolism at 24h, 48h and 72h. In particular, we focused on PC metabolism monitoring changes on total choline (tCho) content represented by GPC plus PCho and free Cho, and on lactate levels.

**Fig 4:**
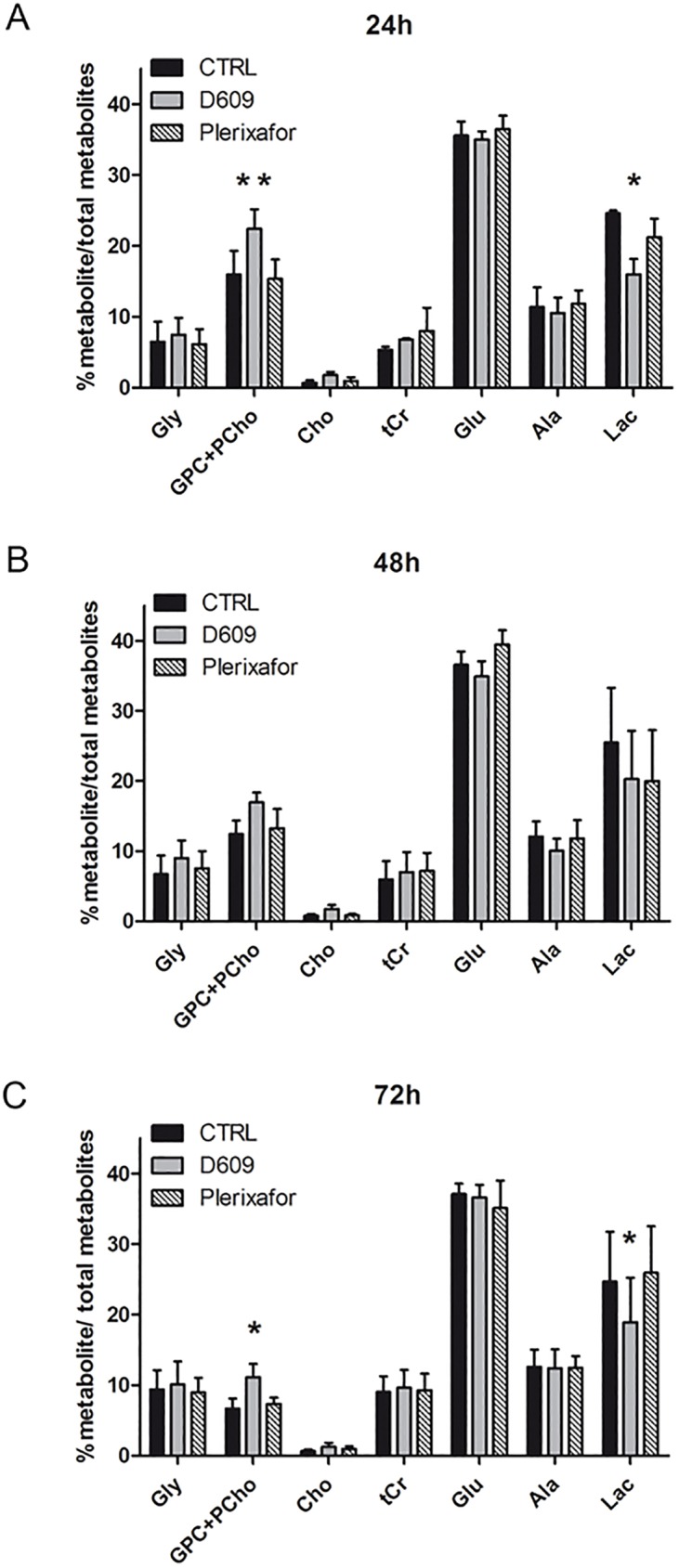
Effects of treatments on U87MG cell metabolism. The histograms represent the means ± SD of percentage values obtained from quantitative ^1^HMRS analysis of each metabolite/percentage of total metabolites content in U87MG untreated (CTRL), treated with D609 or Plerixafor at 24h (A), 48h (B), 72h (C) of treatment. Metabolites reported are the compounds that mainly contribute to the U87MG metabolic profile: Gly, glycine; GPC+PCho, glycerophosphocholine+phosphocholine; Cho, choline; tCr, total creatine (creatine+phosphocreatine); Glu, glutamate; Ala, alanine; Lac, Lactate. Total metabolites = 100%. Means ± SD of n = 3 independent experiments. Statistical analyses were performed with two-tailed unpaired t Student’s test. CTRL versus D609: 24h GPC+PCho **P = 0.0096, Lac *P = 0.0170; 72h GPC+PCho *P = 0.0286, Lac *P = 0.047.

MRS analyses showed a significant increase in the GPC+PCho content in D609-treated cells compared to the corresponding control cells at each time of treatment (Fig 4A–4C) with a significant increase of 35% at 24h and 40% at 72h (Fig 4A and 4C). The increment of GPC plus PCho was entirely due to an increase of GPC, as shown in S4 and S5 Figs (in particular significant 35% increase at 24h and 38% increase at 72h). No significant changes were found in Plerixafor-treated cells (Fig 4A–4C).

Moreover, MRS analysis showed that D609 treatment induced also a significant decrease in the lactate concentration (Lac) at 24h and 72h of D609 exposure (35% and 25% respectively) (Fig 4A and 4C) suggesting that PC-PLC inhibition may interfere with glycolysis in U87MG cells.

**Fig 5:**
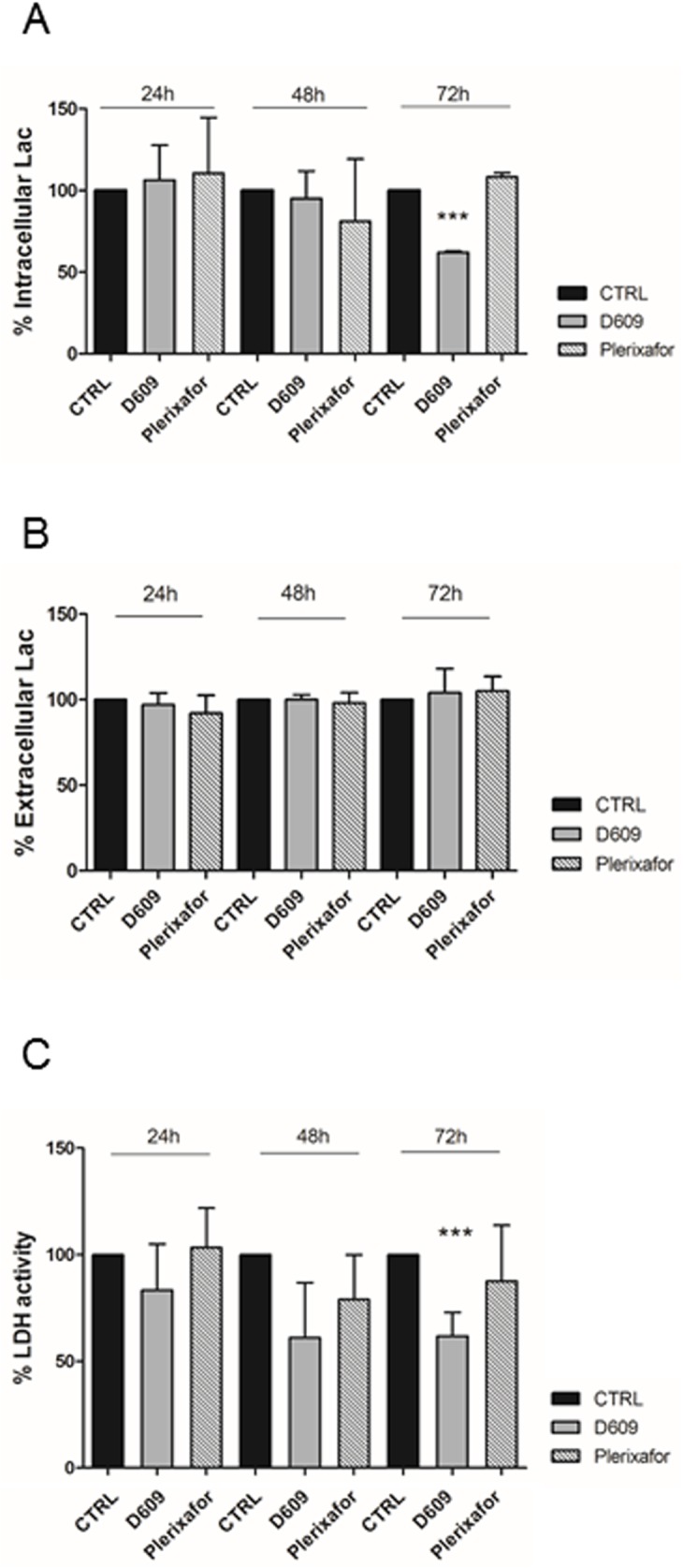
Lactate and LDH measurements in U87MG cells. A) Histogram represents mean values of percentage of intracellular Lac concentration in U87MG obtained colorimetric assay after 24h, 48h, 72h of treatments. Mean value ± SD of n = 3 independent experiments. Statistical analyses were performed using one-way ANOVA. ***P = 0.0003. B) Histogram represents mean ± SD of percentage values (n = 3 independent experiments) of LDH intracellular activity measured by colorimetric assay at different treatment time-points (24h, 48h, 72h). Statistical analyses were performed using one-way ANOVA. ***P = 0.0006; unpaired two-tailed Student-t test CTRL 72h VS D609 72h P = 0.004. C) Histogram represents mean ± SD of percentage values of Lac extracellular concentration measured by a colorimetric assay on U87MG-conditioned medium in response to treatments (24h-48h-72h) (n = 3 independent experiments). All values were normalized for μg of proteins. CTRL values = 100%.

To better understand the effects of D609 on U87MG cells lactate flux, measurements of extracellular and intracellular Lac and Lactate dehydrogenase (LDH) activity were performed. These analyses confirmed a significant reduction of intracellular Lac induced by D609 (Fig 5A). LDH activity was reduced at each time point of D609 treatment with a significant effect at 72h (40% reduction) (Fig 5B). No changes were instead found in the levels of extracellular lactate in cultures of D609-treated cells (Fig 5C).

## Discussion

The CXCR4/CXCL12 molecular axis is involved in numerous responses that drive glioma progression, such as tumor cell proliferation, survival, angiogenesis, invasion and migration [31, 8, 32, 33, 34]. Furthermore, we recently investigated the role of CXCR4 in glioma-microenvironment interactions and demonstrated that reducing CXCR4 expression in human U87MG cells promoted M1 features in microglia/macrophages recruited to the tumor area, creating an environment potentially less favorable for tumor growth [11].

The present study highlights for the first time the existence of an interplay between the chemokine receptor CXCR4 and the PC-PLC enzyme in U87MG human GBM cells. PC-PLC is a catabolic enzyme of the PC cycle, involved in numerous aspects of tumor biology with links to cell signaling and cell membrane receptors status. We reported that PC-PLC inactivation results in the downregulation of some membrane receptors involved in the tumor progression of cancer cells [15, 16, 19, 20].

In the present work we show that PC-PLC co-localizes with the CXCR4 receptor on the plasma membrane of U87MG cells and that these two proteins are co-immunoprecipitated by the respective antibodies. PC-PLC inhibition by D609 down-modulates CXCR4 expression from cell plasma membrane as the well-known CXCR4 antagonist, but the PC-PLC inhibitor is also able to decrease the CXCR4 total protein content [35, 11]. Interestingly, Plerixafor treatment also reduced PC-PLC expression from the U87MG plasma membrane along with the total amount of PC-PLC protein further supporting our data on the existence of an interaction between the two proteins. Furthermore, it is important to note that PC-PLC expression decreases over time in untreated control U87MG cells during the growth curve. As suggested by Fu et al 2009, the expression level and activities of PC-PLC could change in a cell-cycle-dependent manner and were inversely correlated with the expression of Cdc20 [36]. Thus, the basal decrease of PC-PLC content that we observed in our experimental conditions, could be modulated by an intrinsic intracellular machinery responsible for protein turnover, able to guarantee proliferation signaling pathways in these cells.

D609 also caused a strong reduction in U87MG cell invasion potential (down to 60% of control value) and decreased cell proliferation, an effect associated with a reduction in the expression levels of both phosphorylated and total AKT. WB analyses showed that D609 as well as Plerixafor did not induce any effect on ERK expression and activation in U87MG at any analyzed time point. It is worth noting that AKT and ERK1/2 activation by CXCR4 could be independent pathways. In fact, Peng and co-workers demonstrated that AKT activation by CXCR4, did not require ERK1/2 activation in HeLa cells [37]. By using specific inhibitors of p-AKT/AKT and p-ERK/ERK axis the authors observed that CXCR4-activated migration is mediated by AKT but not by ERK. Thus, we hypothesized that the motility reduction we observed in U87MG cells was associated to CXCR4 inactivation by both inhibitors likely mediated by p-AKT/AKT axis. On the other hand, the specific inhibition of CXCR4 by Plerixafor did not affect U87MG cell proliferation, and did not interfere with ERK 1/2 activation, while the inhibition of U87MG cell proliferation by D609 could be partly mediated by p-AKT/AKT axis and other molecular mechanisms, such as inhibition of EGFR activation.

Indeed, we found that the PC-PLC inhibitor was able to down-regulate EGFR and p-EGFR expression. In this context, a previous study by our group [16], focused on HER2 over-expressing breast cancer cells, showed that PC-PLC physically associated with both HER2 and EGFR receptors and its inhibition resulted in a decrease in the overall cellular contents of EGFR, HER2 and HER3 receptors. The down-modulation of both p-EGFR and total EGFR total contents by D609 were also observed in human squamous carcinoma cell lines [19]. These results could be explained by the possible role of D609 as a stress-inducer able to elicit ligand-independent EGFR internalization [38], thus reducing the constitutive expression of the receptor and, likely, to stimulate its degradation. In the present work we showed that in U87MG cells CXCR4-immunoprecipitated proteins were associated with the EGFR receptor, suggesting that the effects observed on EGFR could be linked to the CXCR4-PC-PLC interactions. CXCR4 has been described to transactivate EGFR in breast cancer cells [39] and in ovarian cancer cells in which it leads to both mitogen-activated protein kinase and AKT activation [40]. Our results suggest a possible cross-talk between PC-PLC/CXCR4/EGFR-driven intracellular pathways, in which the PC-PLC protein could act as a molecular adapter link between the two receptors. Thus, the inhibitory effect of D609 on EGFR activation and expression could be associated with the concomitant down-modulation of PC-PLC expression observed in response to D609.

Indeed our previous studies on breast and ovarian cancer cells [16, 20] showed that a physical interaction between an activated PC-PLC enzyme and growth factor receptors, detected by co-immunoprecipitation assays, is crucial to the functional localization of these receptors on plasma membrane [20]. We hypothesize that inhibition of PC-PLC activity can both perturb the phospholipid bilayer and alter key protein-lipid interactions, either by altering the local diacylglycerol (DAG) concentration and/or by interfering with the DAG/phosphatidic acid balance, which is involved in recruiting cytosolic proteins to the membrane and growth signaling potentiation [41]. Furthermore DAG, the substrate of the DAG-Kinase (DAGK) reaction, is a key intracellular signaling factor that activates PKCs and Ras guanyl nucleotide-releasing proteins.

A key message of the present study is that inhibition of PC-PLC by D609 also downmodulates CXCR4, EGFR expression and downstream signaling pathways responsible for cell growth and cell motility in our experimental glioma cells.

Further studies are however needed to identify the molecular domains responsible for PC-PLC/CXCR4/EGFR interactions and the mechanisms involved in D609 effects on EGFR and CXCR4 receptors.

Moreover, interestingly, we observed alterations in U87MG cell metabolism in response to D609. It is well known that cancer cells can reprogram their overall metabolism to facilitate cell growth and survival, through alterations in glucose, glutamine and lipid metabolism [42, 43]. In particular, an increased glucose uptake, low mitochondrial respiration and high aerobic glycolysis, well known as Warburg effect [44], have been reported as the most common metabolic remodeling in tumor cells, including glioma [45–47]. Our experimental model, the U87MG cells, have been described to have a high glycolytic activity, which represents the main part of the total U87MG basal bioenergetic metabolism, contributing to 70% of the total ATP pool [48–50]. Our analysis on MRS metabolic profiles and biochemical assays on U87MG cells highlighted a reduction in intracellular lactate concentration upon D609 treatment together with a decrease in LDH activity, the enzyme that catalyzes the conversion of pyruvate to lactate, suggesting that PC-PLC inhibition hampers with glioma cells glycolysis interfering with the metabolic properties advantageous for GBM progression.

The PI3K pathway has been described in several studies to regulate glucose metabolism in cancer [51, 52], thus our findings on inhibitory effects on CXCR4 expression and EGFR activation/expression could be involved in the reduction of glycolytic flux by the inhibition of PI3K/AKT axis.

We also observed in D609-treated U87MG cells an increase of tCho signal, in particular we found a significant increment of GPC, which represents 80% of tCho in the U87MG basal cell line, as shown in S4 Fig (panel A). The elevated levels of tCho in response to PC-PLC inhibition may be linked to the activation of lipases and phospholipases in response to the treatment that can induce an accumulation of neutral lipids associated with the observed anti-proliferative effect [53].

Further investigations are warranted to better elucidate the mechanisms that underlie PC-PLC/CXCR4 interactions and the metabolic alterations induced by PC-PLC inhibition.

The here reported experimental evidence on the multiple effects exerted by PC-PLC inhibition on CXCR4 expression, signaling and cell metabolism in the human U87MG cells suggests that targeting the PC-PLC enzyme could represent a potential novel strategy for GBM treatment which could contribute to counteract tumor growth and progression by interacting with crucial components of glioma biology such as CXCR4 and EGFR receptors and their downstream effectors.

## Supporting information

S1 Fig Cytostatic effect of D609 on in vitro cultured U87MG cells. Inhibition of cell proliferation induced by the PC-PLC inhibitor D609 (10.0–300.0 μM) on cells treated for 48h. Cell viability, determined using the MTT test (see Materials and Methods) were quantified as percentages of the number of cells in untreated controls at t = 0. The values at each concentration represent the mean (± SD) of 3 independent series of assays. EC50 value = 100 μM.
(TIF)

S2 Fig Immunoprecipitation of CXCR4 with EGFR from U87MG cells. Western blot (WB) assays of proteins isolated from U87MG cells by immunoprecipitation with anti-CXCR4 Ab (IP-α-CXCR4) and blotted with anti-CXCR4 (45 kDa), anti-EGFR (177 kDa) or control IgG heavy chains (*). Right panel represents EGFR and CXCR4 expression in total cell lysate. The central panel shows IP-α-CXCR4blotted with α-EGFR compared to control (CTR IgG) (left panel). *IgG heavy chains.
(TIF)

S3 Fig Effects of treatment on p-ERK and total ERK expression. Representative WB p-ERK and ERK detection in U87MG at 24h, 48h and 72h of treatment with either D609 or Plerixafor. β-actin was used as loading control. The histograms represent the mean values (± SD) of the relative fold changes in p-ERK and total ERK optical density normalized to β-actin, obtained by densitometric analyses of the respective WB protein bands (Image J software). CTRL values = 1. Data were obtained from n = 3 independent experiments.
(TIF)

S4 Fig Quantification of total choline containing metabolites in U87MG cells. A) Representative ^1^H MR spectrum (700 MHz) of aqueous extracts of untreated U87MG cells. Peak assignments: tCr, total creatine (creatine plus phosphocreatine); glx, glutamate plus glutamine; ac, acetate; ala, alanine; lac, lactate; tCho, “total choline-containing compounds”; internal reference signal TSP, trimethylsilylpropanoic acid, a chemical compound containing a trimethylsilyl group, used as reference for aqueous solvents. Expanded ^1^H MRS profiles of tCho region in aqueous extracts (and peak assignment in dashed line) in untreated U87MG cells. Peak assignments: Cho, choline; GPC, glycerophosphocholine; PCho, phosphocholine. The histogram represents percentage of quantitative ^1^HMRS-detected GPC, PCho or Cho contents versus the total amount of total choline (tCho = GPC+PCho+Cho) in the U87MG basal metabolic profile. tCho = 100%. Means ± SD of n = 3 independent determinations. B) Histograms represent means ± SD of percentage values obtained from quantitative ^1^HMRS analysis of the tCho resonance band (GPC, PCho and Cho signals) represented as metabolite/percentage of total metabolites in U87MG untreated (CTRL), D609- or Plerixafor-treated cells analyzed at 24h, 48h, 72h of treatment. Total metabolites = 100%. Means ± SD of n = 2 independent experiments.
(TIF)

S5 Fig Effects of D609 on ^1^H MRS profile in U87MG cells. A) Representative ^1^H MR spectra (400 MHz) of aqueous extracts of 48h of treatment of D609- and untreated control U87MG cells. Peak assignments: tCr, total creatine (creatine plus phosphocreatine); glx, glutamate plus glutamine; ac, acetate; ala, alanine; lac, lactate; tCho, “total choline-containing compounds”.
(TIF)
