# Supplementary figures and images for: Phosphatidylcholine-specific phospholipase C inhibition down- regulates CXCR4 expression and interferes with proliferation, invasion and glycolysis in glioma cells
Source: PLoS One. 2017 Apr 19;12(4):e0176108. doi: 10.1371/journal.pone.0176108 (PMC5397108; doi:10.1371/journal.pone.0176108)

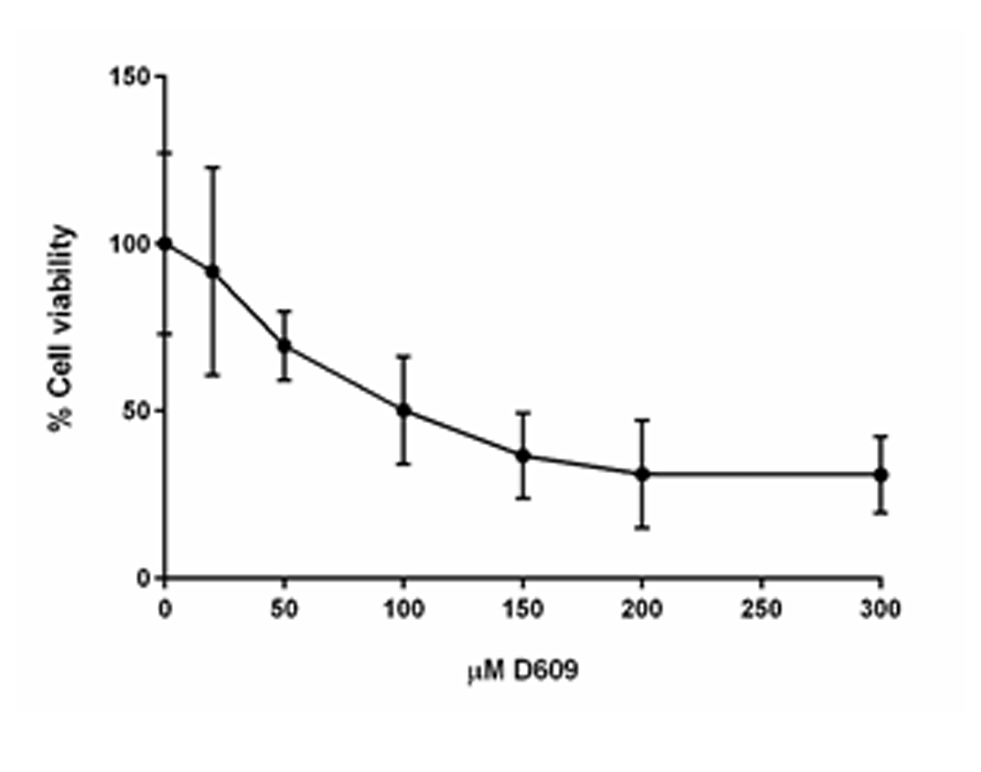

Supplement: S1 Fig — Inhibition of cell proliferation induced by the PC-PLC inhibitor D609 (10.0–300.0 μM) on cells treated for 48h. Cell viability, determined using the MTT test (see Materials and Methods) were quantified as percentages of the number of cells in untreated controls at t = 0. The values at each concentration represent the mean (± SD) of 3 independent series of assays. EC50 value = 100 μM. (TIF) [file pone.0176108.s001.tif]

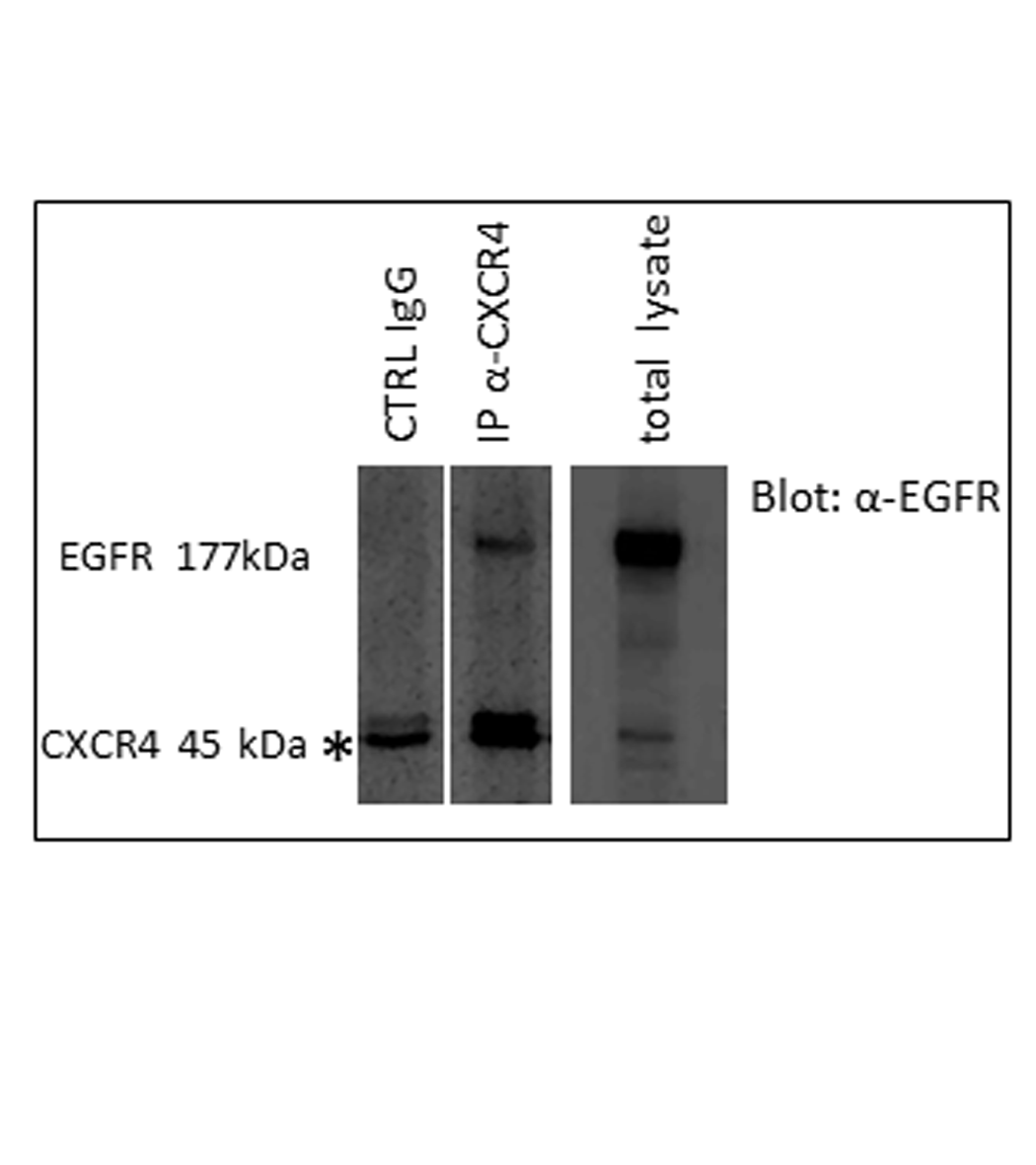

Supplement: S2 Fig — Western blot (WB) assays of proteins isolated from U87MG cells by immunoprecipitation with anti-CXCR4 Ab (IP-α-CXCR4) and blotted with anti-CXCR4 (45 kDa), anti-EGFR (177 kDa) or control IgG heavy chains (*). Right panel represents EGFR and CXCR4 expression in total cell lysate. The central panel shows IP-α-CXCR4blotted with α-EGFR compared to control (CTR IgG) (left panel). *IgG heavy chains. (TIF) [file pone.0176108.s002.tif]

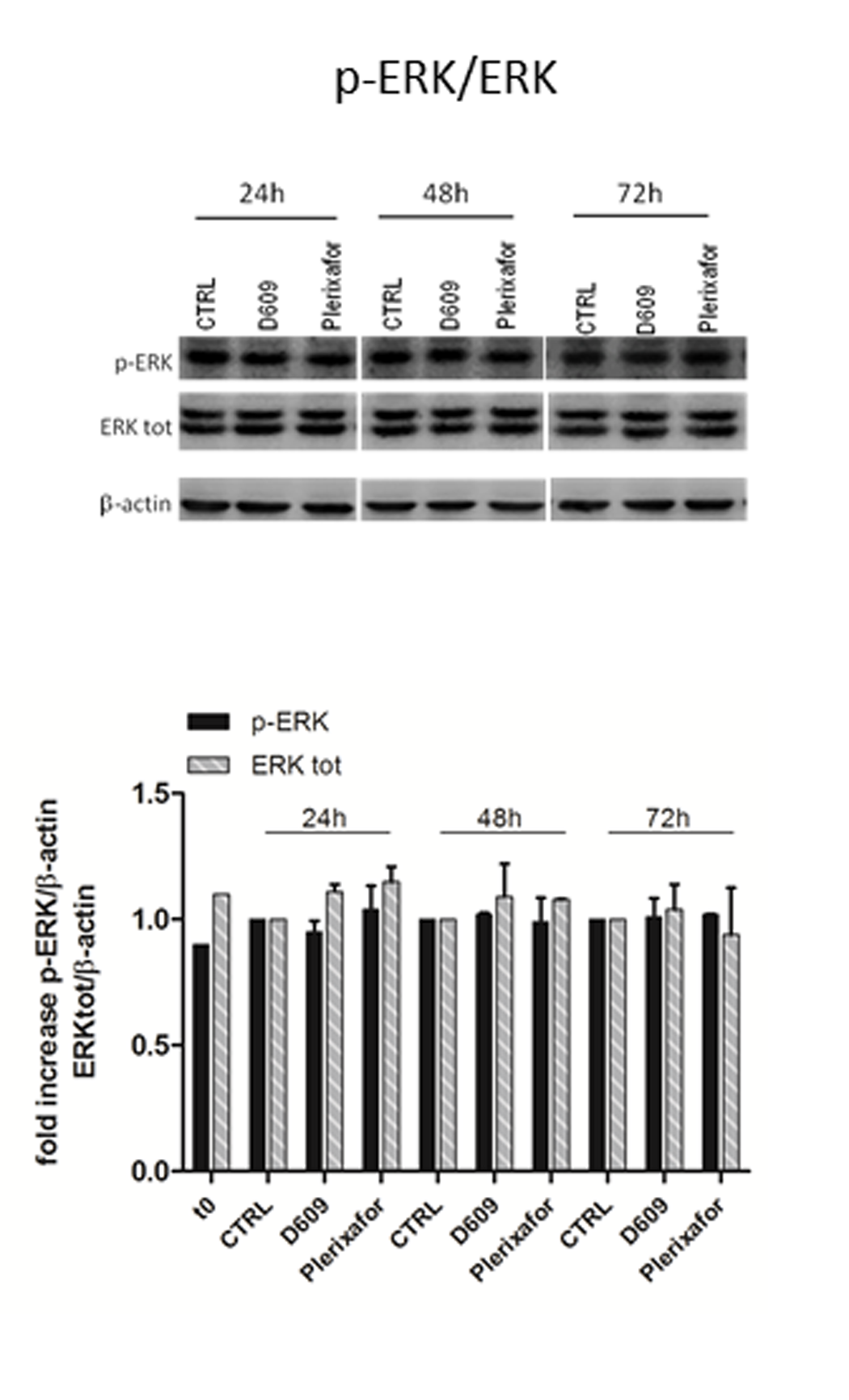

Supplement: S3 Fig — Representative WB p-ERK and ERK detection in U87MG at 24h, 48h and 72h of treatment with either D609 or Plerixafor. β-actin was used as loading control. The histograms represent the mean values (± SD) of the relative fold changes in p-ERK and total ERK optical density normalized to β-actin, obtained by densitometric analyses of the respective WB protein bands (Image J software). CTRL values = 1. Data were obtained from n = 3 independent experiments. (TIF) [file pone.0176108.s003.tif]

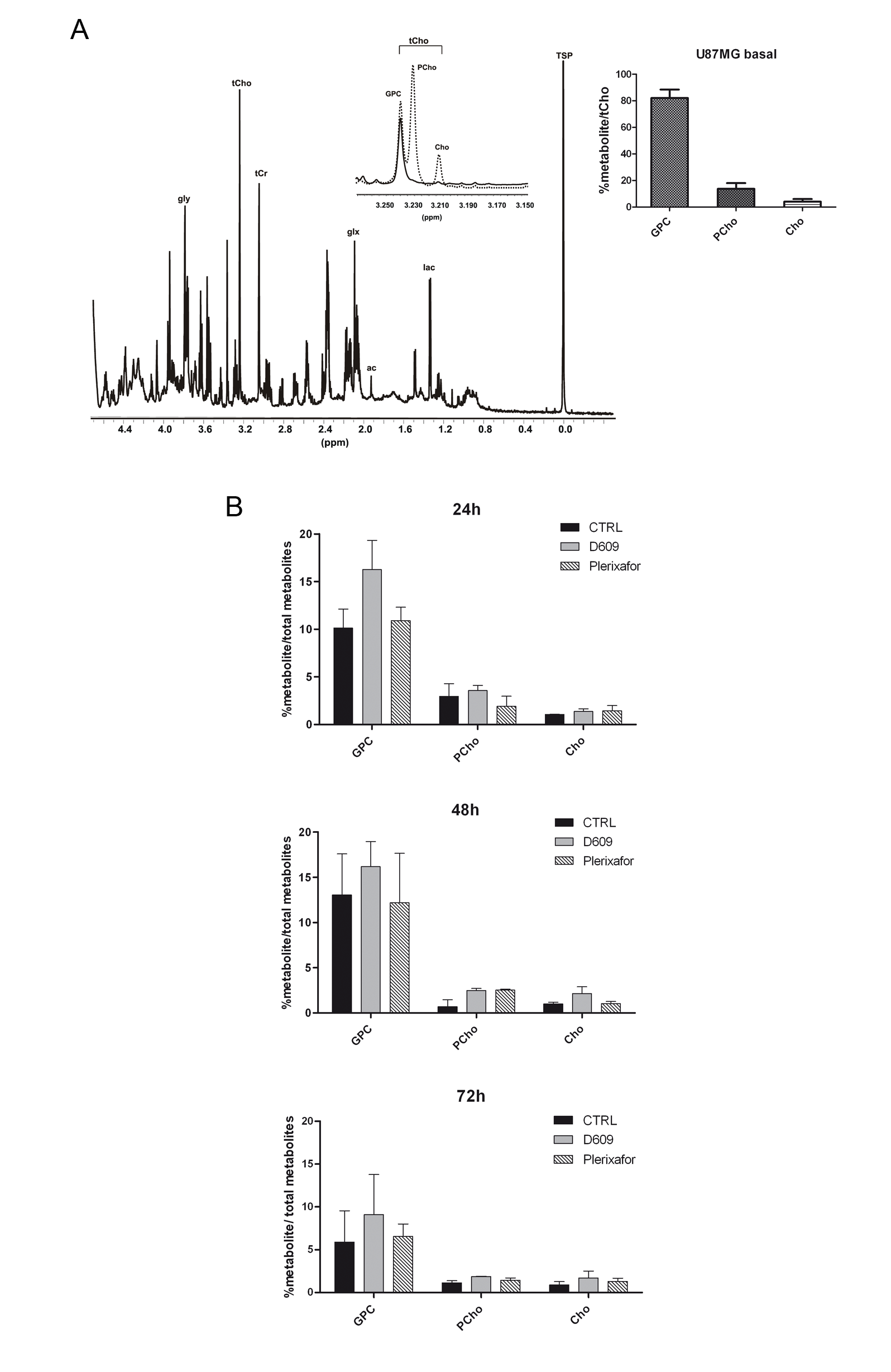

Supplement: S4 Fig — A) Representative 1H MR spectrum (700 MHz) of aqueous extracts of untreated U87MG cells. Peak assignments: tCr, total creatine (creatine plus phosphocreatine); glx, glutamate plus glutamine; ac, acetate; ala, alanine; lac, lactate; tCho, “total choline-containing compounds”; internal reference signal TSP, trimethylsilylpropanoic acid, a chemical compound containing a trimethylsilyl group, used as reference for aqueous solvents. Expanded 1H MRS profiles of tCho region in aqueous extracts (and peak assignment in dashed line) in untreated U87MG cells. Peak assignments: Cho, choline; GPC, glycerophosphocholine; PCho, phosphocholine. The histogram represents percentage of quantitative 1HMRS-detected GPC, PCho or Cho contents versus the total amount of total choline (tCho = GPC+PCho+Cho) in the U87MG basal metabolic profile. tCho = 100%. Means ± SD of n = 3 independent determinations. B) Histograms represent means ± SD of percentage values obtained from quantitative 1HMRS analysis of the tCho resonance band (GPC, PCho and Cho signals) represented as metabolite/percentage of total metabolites in U87MG untreated (CTRL), D609- or Plerixafor-treated cells analyzed at 24h, 48h, 72h of treatment. Total metabolites = 100%. Means ± SD of n = 2 independent experiments. (TIF) [file pone.0176108.s004.tif]

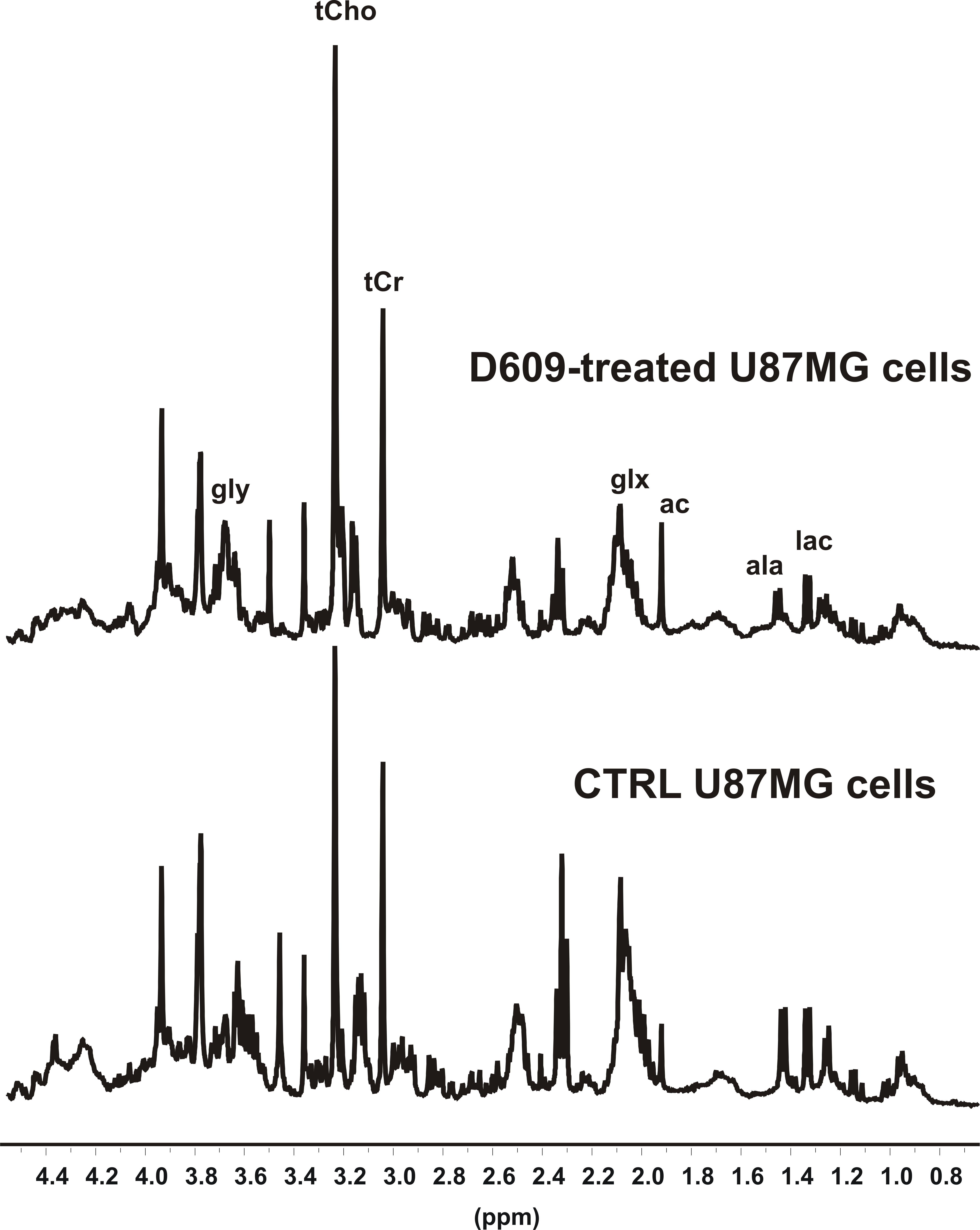

Supplement: S5 Fig — A) Representative 1H MR spectra (400 MHz) of aqueous extracts of 48h of treatment of D609- and untreated control U87MG cells. Peak assignments: tCr, total creatine (creatine plus phosphocreatine); glx, glutamate plus glutamine; ac, acetate; ala, alanine; lac, lactate; tCho, “total choline-containing compounds”. (TIF) [file pone.0176108.s005.tif]
